# CD36 aggravates podocyte injury by activating NLRP3 inflammasome and inhibiting autophagy in lupus nephritis

**DOI:** 10.1038/s41419-022-05179-9

**Published:** 2022-08-23

**Authors:** Fu Lv, Yingxin He, Hongde Xu, Yongchun Li, Lipei Han, Lijie Yan, Hui Lang, Yafei Zhao, Zhanzheng Zhao, Yuanyuan Qi

**Affiliations:** 1 Nephrology Hospital, the First Affiliated Hospital of Zhengzhou University, Zhengzhou University, Henan 450052 China; 2 School of Pharmaceutical Sciences, Zhengzhou University, 100 Ke xue Avenue, Zhengzhou, Henan 450001 China

**Keywords:** Mechanisms of disease, Inflammasome, Lupus nephritis, Autophagy

## Abstract

A major cause of proteinuria in lupus nephritis (LN) is podocyte injury, and determining potential therapeutic targets to prevent podocyte injury is important from a clinical perspective in the treatment of LN. CD36 is involved in podocyte injury in several glomerulopathies and was reported to be a vital candidate gene in LN. Here, we determined the role of CD36 in the podocyte injury of LN and the underlying mechanisms. We observed that CD36 and NLRP3 (NLR family pyrin domain containing 3) were upregulated in the podocytes of lupus nephritis patients and MRL/lpr mice with renal impairment. In vitro, CD36, NLRP3 inflammasome, and autophagy were elevated accompanied with increased podocyte injury stimulated by IgG extracted from lupus nephritis patients compared that from healthy donors. Knocking out *CD36* with the *CRISPR/cas9* system decreased the NLRP3 inflammasome levels, increased the autophagy levels and alleviated podocyte injury. By enhancing autophagy, NLRP3 inflammasome was decreased and podocyte injury was alleviated. These results demonstrated that, in lupus nephritis, CD36 promoted podocyte injury by activating NLRP3 inflammasome and inhibiting autophagy by enhancing which could decrease NLRP3 inflammasome and alleviate podocyte injury.

## Introduction

Systemic lupus erythematosus (SLE) is a chronic and systemic autoimmune disease characterized by heterogeneous manifestations and the production of multiple autoantibodies. Lupus nephritis (LN) is one of the most common and serious complications of SLE. Although studies have emphasized the importance of podocyte injury in lupus glomerulonephritis [1–3], the underlying molecular mechanisms are poorly defined. Therefore, a better understanding of podocyte injury in LN is needed.

CD36 belongs to the scavenger receptor class B (SR-B), a family of highly glycosylated transmembrane receptors with a wide range of ligand recognition sites. Because of its wide range of ligands, CD36 has plenty of functions, including but not limited to recognizing and ingesting lipids, participating in the process of inflammatory response, signal transduction, and apoptosis [4–6]. CD36 is expressed on a wide variety of cell surfaces. In renal tissue, CD36 is mainly expressed in intrinsic cells including podocytes, tubular epithelial cells, and mesangial cells [7]. CD36 promotes the podocyte injury in many kidney diseases including primary nephrotic syndrome [8], obesity-related glomerulopathy [9], and diabetic nephropathy [10]. Moreover, genetic knockout or antagonist blockade of CD36 could alleviate kidney injury indicating that CD36 is a potential therapeutic target [7, 11].

Rare genetic variations in *CD36* were identified in both SLE-pSS families by whole-exome sequencing providing a genetic association between SLE and *CD36* [12]. Recently, Yang et al. raised the possibility that CD36 is the vital candidate gene in pathogenesis of LN using bioinformatics showing that CD36 was significantly upregulated in renal tissues of LN patients and positively associated with the aggravation of LN [13]. However, the molecular mechanisms by which CD36 functions in the pathogenesis of podocyte injury in lupus nephritis remain elusive.

The recognition and binding of ox-LDL by CD36 induce the production of a TLR heterodimer, which further mediates the activation of the NLRP3 (NLR family pyrin domain containing 3) inflammasome [14]. It has been reported that CD36 can activate the NLRP3 inflammasome contributing to the podocyte injury in obesity-related glomerulopathy and primary nephrotic syndrome [8, 9]. However, whether CD36 promotes podocyte injury by regulating the NLRP3 inflammasome in lupus nephritis remains to be established.

The NOD-like receptor 3 (NLRP3) inflammasome is a multimeric protein that contains NLRP3, apoptosis-associated speck-like protein (ASC), and procaspase-1 upon activation promoting inflammation and pyroptotic cell death [15]. Although the underlying mechanisms is still unclear, it has been recognized that autophagy could regulate inflammasome activation. Autophagy is a lysosome-mediated intracellular degradation process and is also important for the regulation of NLRP3 inflammasome activation and elimination. Microtubule-associated protein 1 light chain 3 β (MAP1LC3B) also known as LC3B is the core protein in forming autophagosome and increasing the amplitude of autophagy. The role of autophagy in regulating NLRP3 inflammasome in the podocyte injury in lupus nephritis is less explored.

In this study, we assessed the expression of CD36 in podocytes using renal biopsy samples from lupus nephritis patients as well as renal tissues from MRL/MpJ-Fas<lpr>/J (MRL/lpr) mice. *CD36* knockout podocytes with *CRIPR/cas9* system were used to elucidate the underlying mechanisms of podocyte injury stimulated by IgG extracted from lupus nephritis patients compared with that extracted from healthy donors. Our previous studies have shown that the increased autophagy plays a cytoprotective role in podocyte injury induced by antibodies from LN patients [16]. Therefore, the therapeutic potential of autophagy was also explored in CD36-mediated podocyte injury.

## Methods

### Patients and healthy donors

Blood samples were collected from 75 SLE patients and 24 healthy donors for quantification of gene expression. All the 75 SLE patients, recruited from the First Affiliated Hospital of Zhengzhou University, were fully complied with the American College of Rheumatology revised (ACR) criteria for the classification of SLE, of which 57 were diagnosed with lupus nephritis by renal biopsy (detailed description of the SLE patients were provided in supplementary table1). For immunohistochemistry, 3 renal biopsy specimens from LN patients and 3 paracancerous kidney tissues were collected. Participants provided written informed consent. This study was approved by the Medical Ethics Committee of the First Affiliated Hospital of Zhengzhou University (2019-KY-134).

### Mice

Female MRL/MpJ-Fas<lpr>/J (MRL/lpr) mice were purchased from the Jackson Laboratory (Bar Harbor, ME, USA) and female C57 BL/6 mice were from Liaoning Changsheng Biotechnology Co., Ltd (Liaoning, China). Urine samples from 3 MRL/lpr mice and 3 C57 BL/6 mice were collected at 8, 12, 16, and 20 weeks to quantity proteinuria. For immunohistochemical analysis, western blot, and pathology tests, renal tissues were obtained from 3 MRL/lpr mice and 3 C57 BL/6 mice at 8 and 20 weeks, respectively. All the procedures were approved by the Ethics Committee of the Animal Experimental Center of Zhengzhou University (ZZU-LAC20210604[11]).

### Purification of IgG from sera

Immunoglobulin G (IgG) was extracted from sera of SLE patients and healthy donors using protein G affinity chromatography (17040401, GE Healthcare) as previously described [16]. Then the concentration of IgG was determined by BCA Protein Assay Kit (PT0001, Leagene).

### Podocyte culture and treatments

Immortalized human podocyte (HPC) cell line was kindly provided by Prof Hong Zhang [16]. The HPC was cultured in RPMI-1640 (Biological Industries, USA) supplemented with 10% fetal bovine serum (FBS, Gibco) and Insulin-Transferrin-Selenium (ITS, 41400045, Gibco) at 33 °C in humidified air with 5% CO_2_. To induce HPC differentiation, the cells were transferred to 37 °C for 10 days in the absence of ITS. After podocytes were well differentiated, they were exposed to purified IgG from lupus nephritis patients or healthy donors (stored at −80 °C before use). For inhibition of autophagy, 5 mM 3-methyladenine (3-MA, M9281, Sigma-Aldrich) was added. To induce autophagy, 400 μM rapamycin (RAPA, R8140, Solarbio) was used.

### *CD36* and *MAP1LC3B* knock-out with CRISPR/Cas9

The sgRNA plasmids were designed and constructed by Vigene Biosciences Inc (Shandong, China). The gene sequences for generating sgRNA targeting *CD36* are as follows: sgRNA1 5′-ACGTTAATCTGAAAGGAATC-3′, sgRNA2 5′-GAATCCGACGTTAATCTGAA-3′, sgRNA3 5′-GACAACTATTGTTTCTGCAC-3′. The gene sequences for generating sgRNA targeting *MAP1LC3B* are as follows: sgRNA1 5′-AGATCCCTGCACCATGCCGT-3′, sgRNA2 5′-GAGTTGTGAAGCGCAACCCC-3′, sgRNA3 5′-CCGCAAAACGCATTTGCCAT-3′, sgRNA4 5′-CAAAACGCATTTGCCATCAC-3′, sgRNA5 5′-CCGCCTTTTTGGGTAGAAGT-3′.

These sequences were inserted and cloned into plasmid vector with spCas9 gene and puromycin resistance gene (WZ040004, Vigene). The genomic DNA was extracted and amplified from *CD36* or *MAP1LC3B* knockout podocytes, and PCR sequencing was performed to detect the knock-out. The primers were as follows: hCD36.Fmut 5′-TGTGAGAAGTAACTTGAGTATAAA-3′, hCD36.Rmut 5′-TTTTGGTTGCTAAAGGATT-3′; hMAP1LC3B.Fmut 5′-GCGGGCTGAGGAGATACAAGGG-3′, hMAP1LC3B. Rmut 5′-CTCCCTCGACGGGAAAACCA-3′. Furthermore, the mixed clonal of *CD36* or *MAP1LC3B* knockout podocytes were screened by a limited dilution method to obtain a monoclonal cell line. Finally, the *CD36* knockout (KO) podocyte cell line (*CD36*-KO-HPC) and the *MAP1LC3B* knockout (KO) podocyte cell line (*MAP1LC3B*-KO-HPC) were obtained.

### Gene ORF cDNA clone expression plasmid construction and transfection

The human *CD36* gene coding sequence was ligated into pCMV3-C-EGFP vector (HG10752-ACG, Sino Biological Inc) to construct a *CD36*-overexpression plasmid. The human *MAP1LC3B* gene coding sequence was ligated into the pCMV3-C-EGFP vector (HG14555-ACG, Sino Biological Inc) to construct the *MAP1LC3*-overexpression plasmid. Empty vectors without targeting sequences were used as a negative control (NC). According to manufacturer’s proposal, Lipofectamine 3000 reagent (Invitrogen) was used to transiently transfect the *CD36*-KO-HPC and *MAP1LC3*-KO-HPC cell lines. After 48 h, all the transiently transfected cells were harvested.

### Flow cytometry assay

Podocyte apoptosis was measured by flow cytometry according to the protocol for the Annexin V-PI apoptosis detection kit (556547, BD Biosciences). Then 10,000 cells from each group were passed through and analyzed by a FACScan flow cytometer (Beckman Coulter). The results were analyzed and processed by Flow Jo version 7.6.

### GEO database analysis

In order to obtain the renal transcriptomics data, we searched the data set GSE32591 in the Gene Expression Omnibus (GEO) database. The mRNA expression of *CD36*, *NLRP3*, *CASP1*, *IL1B*, and *NPHS1* in glomeruli was used for comparison between patients with LN and controls.

### Quantification of gene expression

Total RNA from whole blood samples of LN patients (*n* = 57), SLE without LN patients (*n* = 18), and healthy donors (*n* = 24) were extracted and isolated by the TRIzol Reagent (Life Technologies) following the manufacturer’s protocol. Then, the quantification of *CD36* and *NLRP*3 inflammasome-related gene expression was detected by whole genome RNA sequencing (RNA-seq) as previously described [17].

### Western blotting

Proteins were extracted from renal tissues of mice in each group and from human podocytes as mentioned above and processed for Western blotting analysis. We used the following primary antibodies: rabbit anti-CD36 (ab133625, Abcam), rabbit anti-NLRP3 (ab263899, Abcam), rabbit anti-Caspase1 (PA5-87536, Thermofisher), rabbit anti-Cleaved caspase1 (PA5-38099, Thermofisher), rabbit anti-IL-1 beta (ab254360, Abcam), rabbit anti-MAP1LC3B (ab4839, Abcam4), rabbit anti-p62 (ab91526, Abcam), rabbit anti-NPHS2 (ab50339, Abcam), rabbit anti-nephrin (ab58968, Abcam), and rabbit anti-beta-Actin (20536-1-AP, Proteintech). The secondary antibody was a peroxidase-conjugated antibody (IH-0011, DingGuo).

RIPAlysis buffer containing protease inhibitors was used to extract total proteins from frozen tissues or cultured cells. After denaturation with SDS loading buffer, samples containing a fixed concentration of proteins were loaded into an 8–15% SDS–PAGE gel. After electrophoresis, the proteins were transferred to PVDF membranes, which were blocked with 5% skim milk at room temperature for 2 h and incubated with the antibodies mentioned above overnight. The membranes were incubated with the corresponding secondary antibody for 1 h at room temperature. Chemiluminescence assay kit (PK10003, Proteintech) was used to measure protein expression. Images were visualized using a FluorChem R Imaging system (ProteinSimple, USA), and the densitometry quantification was analyzed by Image J version 1.53.

### Immunohistochemical analysis

For immunohistochemistry staining, renal tissues from humans and mice (MRL/lpr and C57 BL/6 mice) were incubated with primary antibodies against CD36 (ab133625, Abcam) and NLRP3 (ab263899, Abcam) and a secondary peroxidase-conjugated antibody (IH-0011, DingGuo) according to the manufacturer’s instructions. Citrate antigen retrieval solution was used for antigen retrieval, endogenous peroxidase was blocked with 3% H_2_O_2_ for 30 min, and nonspecific binding sites were blocked with normal goat serum for 20 min. Primary antibodies were added dropwise and incubated overnight at 4 °C. A peroxidase-conjugated anti-rabbit antibody was used for secondary detection, and then, the signal was developed with a DAB Substrate Kit (DA1010, Solarbio). The sections were observed and imaged using the machine described above. Finally, the collected images were processed by Image J version 1.53.

### Measuring of murine urinary protein levels

Beginning at the age of 8 weeks, female MRL/lpr and C57 BL/6 mice were placed in metabolic cages every four weeks (8, 12, 16, 20 weeks) to collect 24-h urine samples; these samples were sent to the nephropathy laboratory of the First Affiliated Hospital of Zhengzhou University for urinary protein level measurement.

### Statistical analysis

The statistical analyses were performed using SPSS version 21.0. All the values are presented as the mean ± standard deviation. Two-group comparisons were performed by two-tailed Student’s *t* test, and correlations between the parameters were assessed by Pearson correlation analysis. The difference was considered statistically significant when the *p* value was <0.05.

## Results

### The expression of CD36 was upregulated and positively correlated with NLRP3 inflammasome activation in LN patients

**Fig. 1:**
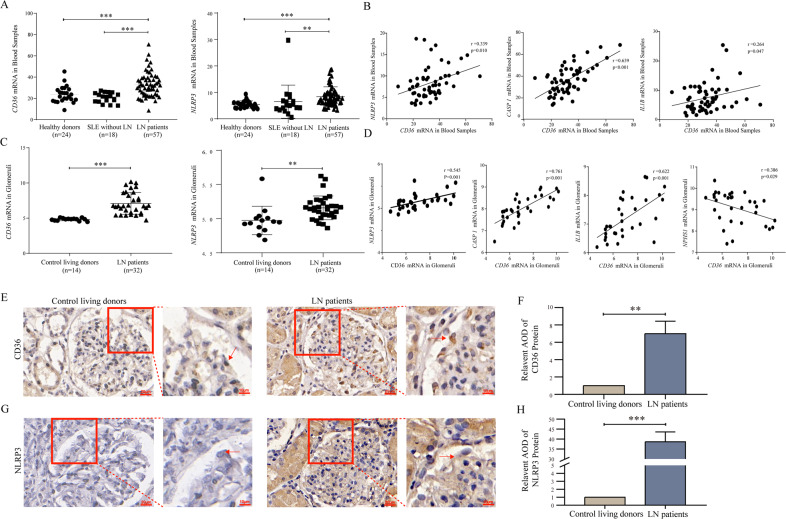
The expression of CD36 was upregulated and positively correlated with NLRP3 inflammasome activation in LN patients. **A** Scatter plot of *CD36* and *NLRP3* mRNA expression in whole blood samples from healthy donors (*n* = 24), SLE without LN (*n* = 18), and LN patients (*n* = 57). **B** Pearson correlation analysis between *CD36* and *NLRP3*, *CASP1*, *IL1B* mRNA expression in whole blood samples. **C** Scatter plot of *CD36* and *NLRP3* mRNA expression in renal glomeruli from Control living donors (*n* = 14) and LN patients (*n* = 32), relevant data are from GEO database (GSE35291). **D** Pearson correlation analysis between *CD36* and *NLRP3*, *CASP1*, *IL1B*, *NPHS1* mRNA expression in renal glomeruli, relevant data are from GEO database (GSE35291). **E**–**H** Immunohistochemical staining showed the localization and relative quantification of CD36, NLRP3 protein in renal biopsy specimens of human (scale bar 20 μm and 10 μm). The relative expression of protein was indirectly reflected by AOD value. The red arrow denoted the most prominent podocyte (original magnification: ×400). Data are expressed as the mean ± SD. **p < 0.01. ***p < 0.001.

The results from whole genome RNA sequencing (RNA-seq) of whole blood samples showed that the expression of *CD36* was significantly upregulated (*p* < 0.001) in LN patients compared with SLE patients without renal impairment or healthy donors (Fig. 1A). Moreover, the levels of *CD36* were also significantly positively associated with the levels of *NLRP3* (*r* = 0.339, *p* < 0.05), *CASP1* (*r* = 0.639, *p* < 0.001) and *IL1B* (*r* = 0.264, *p* < 0.05) in LN patients (Fig. 1B).

To further analyze the expression of *CD36* in renal tissues from LN patients, we searched the GEO database. The gene expression profile from GSE32591 revealed that the expression of *CD36* was significantly higher in the glomeruli of LN patients than in those of controls (*p* < 0.001) (Fig. 1C), and there was a negative correlation between glomerular *CD36* and *NPHS1* (nephrin) mRNA levels (*r* = −0.386, *p* < 0.05), which suggested that the upregulation of CD36 might be correlated with renal podocyte injury (Fig. 1D). It was also observed that the levels of NLRP3 inflammasome components were upregulated in the glomeruli of LN patients and that *CD36* expression was positively correlated with *NLRP3* expression (*r* = 0.545, *p* < 0.01), *CASP1* (*r* = 0.761, *p* < 0.001), and *IL1B* (*r* = 0.622, *p* < 0.001) (Fig. 1D).

Further, we used immunohistochemistry to detect whether CD36 was upregulated in the podocytes of LN patients. The renal biopsy samples from 3 LN patients and 3 paracancerous renal tissues showed that the average optical density (AOD) of CD36-positive products was significantly increased in patients with LN (*p* < 0.01) (Fig. 1E, F). A significant upregulation of NLRP3 was also observed in the podocytes of LN patients (*p* < 0.001) (Fig. 1G, H). Above findings indicated that CD36 may play an important role in the podocyte injury of LN patients via activation of the NLRP3 inflammasome.

### The expression of CD36 and NLRP3 inflammasome was upregulated in MRL/lpr mice with renal impairment

**Fig. 2:**
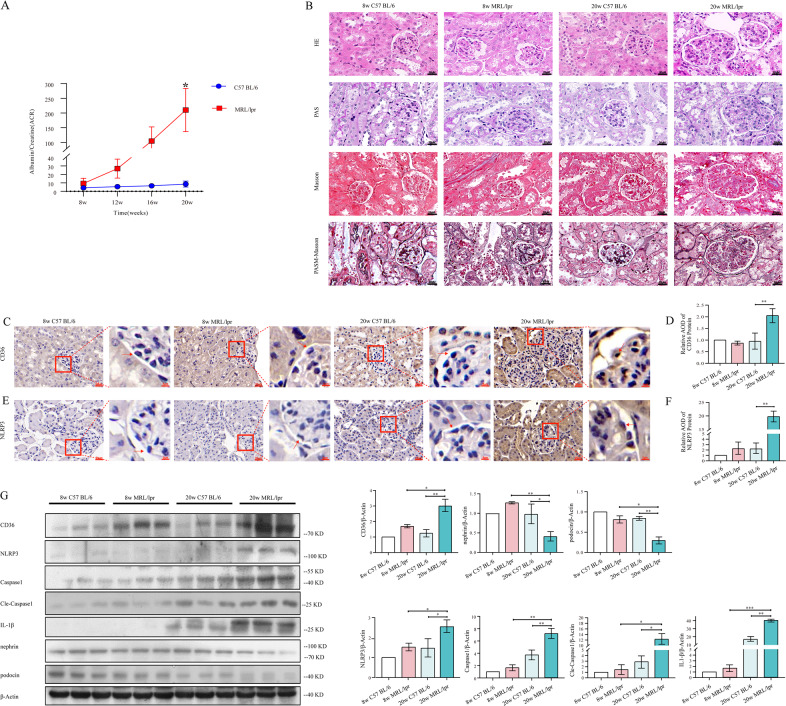
The expression of CD36 and NLRP3 inflammasome was upregulated in MRL/lpr mice with renal impairment. **A** The urinary protein levels of C57 BL/6 and MRL/lpr mice were shown by urinary Albumin/Creatinine (uACR) (20w C57 BL /6 vs. 20w MRL/lpr). **B** Renal tissue sections were stained with HE, PAS, Masson, PASM-Masson to assess renal pathology (scale bar 20 μm) (original magnification: ×400). **C**–**F** Immunohistochemical staining showed the localization and relative quantification of CD36, NLRP3 protein in murine kidney (C57 BL/6 and MRL/lpr mice; scale bar 20 μm and 5 μm). The relative expression of protein was indirectly reflected by AOD value. The red arrow denoted the most prominent podocyte (original magnification: ×400). **G** Quantitative expression of CD36, NLRP3, Caspase1, Cleaved-Caspase1, IL-1β, Nephrin, Podocin, protein in renal tissue homogenate from C57 BL/6 and MRL/lpr mice was detected by western blot analysis (8w MRL/lpr vs. 20w MRL/lpr; 20w C57 BL/6 vs. 20w MRL/lpr). Cle-Caspase1: Cleaved-Caspase1. Data are expressed as the mean ± SD. *p < 0.05. **p < 0.01. ***p < 0.001.

We further validated our above discovery in MRL/lpr mice as well as C57 BL/6 mice as control group. The urine-protein detection showed that urinary albumin/creatinine ratio (uACR) of MRL/lpr mice was significantly increased at 20 weeks (*p* < 0.05) (Fig. 2A) and the renal impairment was confirmed by HE, PAS, Masson and PASM-Masson staining (Fig. 2B). Hence, 8-week and 20-week mice were used for comparison in follow-up experiments.

The average optical density (AOD) of CD36-positive and NLRP3-positive products in renal tissues of 20-week MRL/lpr mice was significantly higher than that of 20-week C57 BL/6 mice (Fig. 2C–F). Western blotting, it was also observed that CD36, NLRP3, Caspase1, Cleaved-Capase1, and IL-1β (interleukin 1 beta) expression was significantly upregulated in 20-week MRL/lpr mice compared with 8-week MRL/lpr mice or 20w C57 BL/6 mice (Fig. 2G).

### Upregulated CD36 induced podocyte injury by activating the NLRP3 inflammasome

To further elucidate the role of CD36 in LN podocyte injury, we treated human podocytes with IgG extracted from LN patients (IgG-LN) or healthy donors (IgG-Control) as the deposition of autoantibodies in glomeruli is the major cause of renal impairment in SLE.

**Fig. 3:**
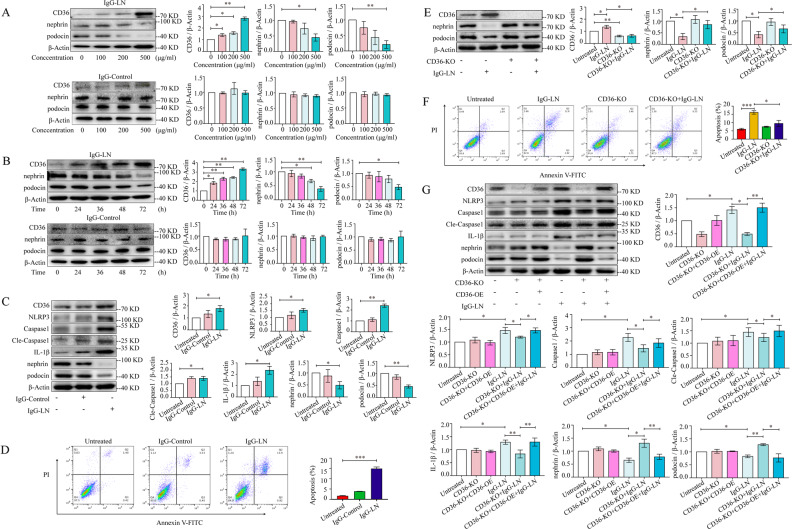
Upregulated CD36 induces podocyte injury via activating NLRP3 inflammasome. **A** Western blot analysis of CD36, Nephrin, and Podocin in human podocytes at different IgG-LN and IgG-Control concentrations (exposure to IgG-LN for 72 h). **B** Western blots analysis of CD36, Nephrin, and Podocin in human podocytes after exposure to 0.5 μg/ml IgG-LN and IgG-Control at various time points. **C** Western blot analysis of CD36, Nephrin, Podocin, NLRP3, Caspase1, Cleaved-Caspase1, and IL-1β in human podocytes with treatment of IgG-LN (500 μg/ml; 72 h) (Untreated vs. IgG-LN). **D** Representatives of Annexin V-PI double-staining in different groups and quantitative of apoptosis was shown in histogram with mean ± SD from 3 experiments. (Untreated vs. IgG-LN). **E** The effect of *CD36* knockout on LN-related podocyte injury was explored by *CRISPR/Cas9* technology and subsequent western blot analysis (Untreated vs. IgG-LN; IgG-LN vs. *CD36* KO + IgG-LN). **F** Representatives of Annexin V - PI double-staining in different groups and quantitative of apoptosis was shown in histogram with mean ± SD from 3 experiments (Untreated vs. IgG-LN; IgG-LN vs.*CD36* KO + IgG-LN). **G** Western blot analysis of CD36, Nephrin, Podocin, NLRP3, Caspase1, Cleaved-Caspase1, and IL-1β in human podocytes under different treatments (Untreated vs. IgG-LN; IgG-LN vs. *CD36* KO + IgG-LN; *CD36* KO + IgG-LN vs. *CD36* KO + *CD36* OE + IgG-LN). Cle-Caspase1: Cleaved-Caspase1. Data are expressed as the mean ± SD. *p < 0.05. **p < 0.01. ***p < 0.001.

Podocytes exposed to IgG-LN exhibited significantly decreased nephrin and podocin levels in a time- and dose-dependent manner compared to podocytes exposed to IgG-Control; this result indicated the effective induction of podocyte injury (Fig. 3A, B). Simultaneously, the levels of CD36 were also elevated in a time- and dose-dependent manner (Fig. 3A, B). For the following experiments, we exposed human podocytes to 500 μg/ml IgG-LN or IgG-Control for 72 h in vitro.

The average rate of podocyte apoptosis was significantly increased when the cells were exposed to 500 μg/ml IgG-LN for 72 h compared with IgG-Control treatment or no treatment (Fig. 3D). Knocking out *CD36* with the *CRISPR/Cas9* system alleviated podocyte injury by reducing the apoptosis rate and increasing nephrin and podocin expression under conditions of IgG-LN exposure (Fig. 3E, F), and podocyte injury was observed after *CD36* overexpression in the *CD36*-knockout podocyte cell line (Fig. 3G). Our results indicated that upregulated CD36 promoted podocyte injury in lupus nephritis.

We also investigated whether the NLRP3 inflammasome was involved in CD36-induced podocyte injury. The expression of NLRP3, Caspase1, Cleaved Caspase1, and IL-1β was also significantly upregulated after exposure to IgG-LN (Fig. 3C). The levels of NLRP3, Caspase1, Cleaved Caspase1, and IL-1β were decreased after knocking out *CD36* but increased after *CD36* overexpression, indicating that the NLRP3 inflammasome mediated the CD36-induced podocyte injury in LN (Fig. 3G).

### Enhancing autophagy reduced NLRP3 inflammasome activation and podocyte injury

**Fig. 4:**
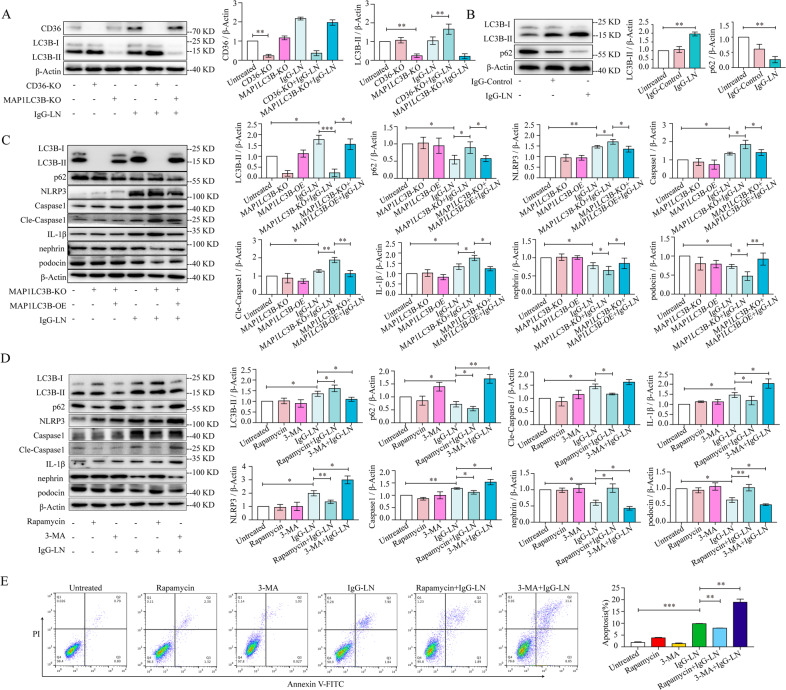
Enhancing autophagy could reduce NLRP3 inflammasome activation and podocyte injury. **A** The regulatory relationship between *CD36* and *MAP1LC3B* was explored by *CRISPR/Cas9* technology and subsequent western blot analysis. **B** Western blotting of autophagy markers LC3B and p62 in human podocytes with treatment of IgG-LN (500 μg/ml; 72 h), compared to no treatment (Untreated vs. IgG-LN). **C** Western blot analysis of LC3B, p62, Nephrin, Podocin, NLRP3, Caspase1, Cleaved-Caspase1, and IL-1β in human podocytes under different treatments (Untreated vs. IgG-LN; IgG-LN vs. *MAP1LC3B* KO + IgG-LN; *MAP1LC3B* KO + IgG-LN vs. *MAP1LC3B* KO + *MAP1LC3B* OE + IgG-LN). **D** Western blot analysis of LC3B, p62, Nephrin, Podocin, NLRP3, Caspase1, Cleaved-Caspase1, and IL-1β in human podocytes under different treatments (Untreated vs. IgG-LN; IgG-LN vs. rapamycin + IgG-LN; IgG-LN vs. 3-MA + IgG-LN). **E** Representatives of Annexin V - PI double-staining in different groups and quantitative of apoptosis was shown in histogram with mean ± SD from 3 experiments (Untreated vs. IgG-LN; IgG-LN vs. rapamycin + IgG-LN; IgG-LN vs. 3-MA + IgG-LN). Cle-Caspase1: Cleaved-Caspase1. Data are expressed as the mean ± SD. *p < 0.05. **p < 0.01. ***p < 0.001.

Then, we constructed *CD36*-KO-HPC and *MAP1LC3B*-KO-HPC cell lines with the *CRISPR/Cas9* system to explore the regulatory relationship between CD36 and MAP1LC3B. Our results showed that the expression of MAP1LC3B significantly increased when *CD36* was knocked out, while the expression of CD36 remained unchanged after knocking out *MAP1LC3B;* these results indicated an inhibitory effect of CD36 on MAP1LC3B (Fig. 4A).

MAP1LC3B is a core component in autophagy and our previous study revealed its protective role in IgG-induced podocyte injury in LN [16]. Autophagy has been reported to play a significant role in the regulation of the NLRP3 inflammasome [18]. In the present study, we explored the relationship between autophagy and the NLRP3 inflammasome in podocyte injury. As shown in Fig. 4, autophagy was induced in podocytes exposed to IgG-LN (Fig. 4B). The expression of NLRP3, Caspase1, Cleaved Caspase1, and IL-1β was increased, while podocin and nephrin levels were decreased after knocking out *MAP1LC3B;* however, the gene expression pattern was restored when *MAP1LC3B* was overexpressed in the *MAP1LC3B*-knockout podocyte cell line (Fig. 4C).

We further treated podocytes with the autophagy activator rapamycin and the autophagy inhibitor 3-MA. It was observed that the expression of NLRP3, Caspase1, Cleaved-Caspase1, IL-1β, and apoptosis was increased, while podocin and nephrin levels were decreased after treatment with 3-MA. The apoptosis and the expression patterns were restored after treatment with rapamycin (Fig. 4D, E).

These results indicated that CD36 could inhibit autophagy and that enhancing autophagy could alleviate podocyte injury by clearing the NLRP3 inflammasome.

## Discussion

It has been well documented that CD36 plays a key role in maintaining lipid and glucose metabolism [19, 20]. However, its role in autoimmune disease has been poorly defined. Studies have shown that CD36 can also recognize and bind to dead cells (apoptosis or necrosis) [21–23]. Dead cells can release autoantigens that disrupt immune tolerance, and such antigens are absolute targets in the pathogenesis of autoimmune diseases, such as SLE [24–26]. Recently, weighted gene co-expression network analysis (WGCNA) suggested that CD36 was a biomarker and a potential therapeutic target of LN [13]. Nonetheless, the precise mechanisms by which CD36 functions in LN podocyte injury remain unknown. The expression of *CD36* was upregulated in glomeruli from LN patients compared with controls [27]. Higher expression of CD36 in podocytes of LN patients and MRL/lpr mice with renal impairment was confirmed by immunohistochemical staining. Moreover, elevated expression of *CD36* was positively correlated with *NLRP3*, *CASP1*, and *IL1B* expression and negatively correlated with *NPHS1* expression in the glomeruli from patients with LN, according to the data from the GEO database (GSE32591) [27]. Furthermore, our findings revealed for the first time that CD36 was upregulated in podocytes from patients with lupus nephritis and might promote podocyte injury by activating the NLRP3 inflammasome.

In our present study, we observed that upon stimulation with IgG extracted from lupus nephritis patients, CD36 was upregulated in podocytes, and NLRP3 inflammasome levels and podocyte apoptosis were increased in vitro. The level of the NLRP3 inflammasome was not increased, and podocyte injury was alleviated after *CD36* knockout, and these phenomena could be reversed after *CD36* overexpression. The inflammation associated with lupus nephritis occurs in response to sterile stimuli, such as autoantibodies. Related studies have shown that inflammation can be triggered by the recognition of sterile damage-associated molecular patterns (DAMPs) via their interactions with pattern recognition receptors (PRRs), such as NOD-like receptors (NLRs), which results in the production of inflammatory cytokines and chemokines [28–30]. Among the NLRs, NLRP3 has been widely studied. After NLRP3 is activated, downstream molecules are recruited and assembled to form the NLRP3 inflammasome. Studies have shown that the NLRP3 inflammasome is overactivated in lupus nephritis, which promotes the exacerbation of inflammation and the progression of LN [29, 31]. Based on previous findings, our results suggested that CD36 promotes podocyte injury by activating the NLRP3 inflammasome in lupus nephritis.

Although our previous findings underlined the protective role of autophagy in LN podocyte injury, detailed mechanisms remain to be determined. In the present study, NLRP3 inflammasome and podocyte injury were alleviated by treatment with an autophagy activator (rapamycin) and were significantly exacerbated when *MAP1LC3B* was knocked out or when an autophagy inhibitor (3-MA) was added. In addition, in the *MAP1LC3B*-KO HPC cell line, overexpression of *MAP1LC3B* decreased inflammation and podocyte injury to the level observed in the control cells. Stimulating autophagy tempered inflammation by eliminating the activity of Caspase-1 and the maturation of IL-1β, but blocking autophagy potentiated inflammasome activity [32]. Consistent with previous findings in macrophages, our study demonstrated that activation of autophagy could alleviate inflammation by reducing NLRP3 inflammasome activation in podocytes.

More importantly, our study also revealed the inhibitory effect of CD36 on MAP1LC3B (an essential gene for autophagy, particularly macroautophagy and *MAP1LC3B*-dependent phagocytosis), the underlying mechanism of which seems to be multifactorial. It was reported that AMP-activated protein kinase (AMPK) was activated and that the expression of ULK1 was also upregulated in CD36-knockdown hepatocytes [33]. Atg1/ULK1 are central components in the induction of autophagy [34]. AMPK could promote autophagy in multiple cells and could also inhibit MTOR function. Our previous study revealed that MTOR was downregulated and autophagy was increased after treatment with IgG extracted from LN patients [16]. It was probable that AMPK activation and MTOR inhibition might promote autophagy in CD36-knockout podocytes. Therefore, for the first time, our study demonstrated that CD36 negatively regulated autophagy in podocytes. CD36 deficiency enhanced autophagy, which reduced podocyte injury by eliminating the NLRP3 inflammasome. The putative effect of AMPK and MTOR on autophagy in CD36-deficient podocytes requires further investigation.

Lipids and high glucose levels can upregulate CD36 expression [35–37]. Much of the available research of CD36 focuses on obesity-related glomerulopathy [9], diabetic nephropathy [10]. Recent study revealed that CD36 might also take part in the lipid disorders in the glomerular tissue of lupus nephritis by weighted gene co-expression network analysis (WGCNA) of GSE104948 from the GEO database [13]. Ectopic fat accumulation in kidney was related with lipotoxicity leading to kidney impairment [38]. Whether the aberrant expression of CD36 contributes to the lipotoxicity damage in lupus nephritis deserves further study.

**Fig. 5:**
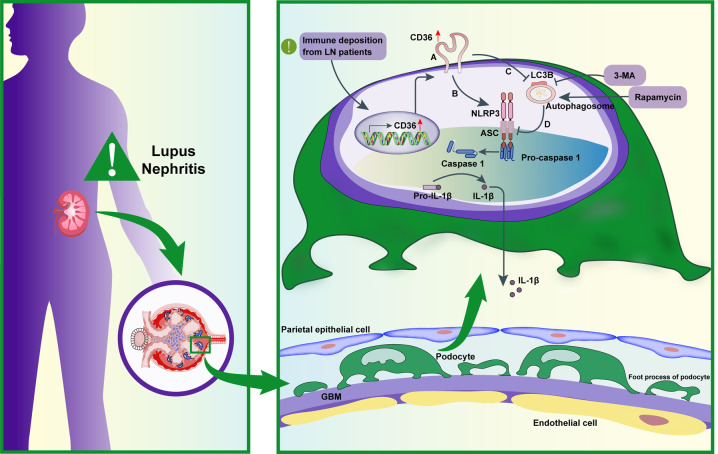
Mechanistic diagram. **A** CD36 was upregulated in the podocyte of lupus nephritis. **B** Elevated CD36 promoted NLRP3 inflammasome and podocyte injury. **C** CD36 negatively regulated autophagy. **D** Enhancing autophagy could reduce NLRP3 inflammasome activation and podocyte injury.

In conclusion (Fig. 5), our study demonstrated that CD36 promoted podocyte injury in lupus nephritis by activating the NLRP3 inflammasome and inhibiting autophagy. By enhancing autophagy, the NLRP3 inflammasome levels were decreased, and podocyte injury was alleviated.

## Supplementary information

supplementary table

full length western blots

checklist

## Data Availability

The data that support the findings of this study are available from the corresponding author upon reasonable request.
