# Supplementary material for: CD36 aggravates podocyte injury by activating NLRP3 inflammasome and inhibiting autophagy in lupus nephritis
Source: Cell Death Dis. 2022 Aug 23;13(8):729. doi: 10.1038/s41419-022-05179-9 (PMC9399182; doi:10.1038/s41419-022-05179-9)
Supplement: Supplementary file 1 — supplementary table [file 41419_2022_5179_MOESM1_ESM.docx]

**CD36 Aggravates Podocyte Injury by Activating NLRP3 Inflammasome and Inhibiting Autophagy in Lupus Nephritis**

Fu Lv^1#^, Yingxin He^2#^, Hongde Xu^2^, Yongchun Li^2^, Lipei Han^1^, Lijie Yan^2^, Hui Lang^2^, Yafei Zhao^1^, Zhanzheng Zhao^1*^, Yuanyuan Qi^1*^

**AUTHORS’ INSTITUTION AND AFFILIATION**

1. Nephrology Hospital, the First Affiliated Hospital of Zhengzhou University, Zhengzhou University, Henan 450052, China;
2. School of Pharmaceutical Sciences, Zhengzhou University, 100 Ke xue Avenue, Zhengzhou, Henan 450001, China.

^#^: These authors contribute equally to this work.

CORRESPONDING AUTHORS

Dr. Zhan-zheng Zhao, MD & PhD;

Email: zhanzhengzhao@zzu.edu.cn

Nephrology Hospital, the First Affiliated Hospital of Zhengzhou University,

Institute of Nephrology, Zhengzhou University

No.1, Jianshe Road, Erqi District

Zhengzhou 450052, P.R China

Dr. Yuan-yuan Qi, MD & PhD;

Email: qqyyiillyy@126.com

Nephrology Hospital, the First Affiliated Hospital of Zhengzhou University,

Institute of Nephrology, Zhengzhou University

No.1, Jianshe Road, Erqi District

Zhengzhou 450052, P.R China

Supplementary table 1. Clinical manifestations of SLE patients.

| Clinical manifestations | Quantification of gene expression | | |  | Immunohistochemistry |
| --- | --- | --- | --- | --- | --- |
|  | SLE patients (without renal impairment) (n=18) | LN patients (n=57) | SLE patients (n=75) |  | LN patients (n=3) |
| Gender (M/F) | 2/16 | 10/47 | 12/63 |  | 0/3 |
| Onset age (years) (mean ± SD) | 30.65±11.61 | 30.4±11.93 | 30.46±11.86 |  | 24, 38, 53* |
| Malar rash (+, %) | 7 (38.9) | 2 (3.5) | 9 (12) |  | 0 (0) |
| Discoid rash (+, %) | 2 (11.1) | 0 (0) | 2 (2.7) |  | 0 (0) |
| Photosensitivity (+, %) | 0 (0) | 1 (1.8) | 1 (1.3) |  | 0 (0) |
| Oral ulcers (+, %) | 0 (0) | 0 (0) | 0 (0) |  | 0 (0) |
| Nonerosive arthritis (+, %) | 1 (5.6) | 3 (5.3) | 4 (5.3) |  | 1 (33.3) |
| Pleuritis or pericarditis (+, %) | 1 (5.6) | 12 (21.1) | 13 (17.3) |  | 1 (33.3) |
| Renal disorder |  |  |  |  |  |
| Serum creatinine (μmol/L) (median, IQR) | 55.50  (42.25-64.40) | 65.00  (56.00-106.00) | 63.00  (56.00-86.00) |  | 54, 85, 88* |
| Urinary protein (g/24 hours) (median, IQR) | / | 0.47  (0.14-1.57) | / |  | 1.33, 1.82, 0.26* |
| Pathological classification (+, %) |  |  |  |  |  |
| II | / | 5 (8.77) | / |  | 0 (0) |
| III | / | 13 (22.81) | / |  | 2 (66.7) |
| IV | / | 21 (36.84) | / |  | 0 (0) |
| V | / | 5 (8.77) | / |  | 1 (33.3) |
| III+V | / | 5 (8.77) | / |  | 0 (0) |
| IV+V | / | 8 (14.04) | / |  | 0 (0) |
| Neurologic disorder (+, %) | 0 (0) | 1 (1.8) | 1 (1.3) |  | 0 (0) |
| Hematological disorder (+, %) |  |  |  |  |  |
| Hemolytic anemia | 0 (0) | 0 (0) | 0 (0) |  | 0 (0) |
| Leukopenia | 6 (33.3) | 2 (3.5) | 8 (10.7) |  | 0 (0) |
| Lymphopenia | 8 (44.4) | 2 (3.5) | 10 (13.3) |  | 1 (33.3) |
| Thrombocytopenia | 2 (11.1) | 5 (8.8) | 7 (9.3) |  | 0 (0) |
| Immunologic disorder |  |  |  |  |  |
| Anti-dsDNA (+, %) | 5 (27.8) | 16 (28.1) | 21 (28) |  | 0 (0) |
| Anti-Sm (+, %) | 4 (22.2) | 7 (12.3) | 11 (14.7) |  | 1 (33.3) |
| C3 (g/L) (mean ± SD) | 0.89±0.24 | 0.96±0.32 | 0.95±0.31 |  | 1.19, 1.58, 0.98* |
| C4 (g/L) (mean ± SD) | 0.15±0.08 | 0.22±0.09 | 0.21±0.1 |  | 0.21, 0.40, 0.32* |
| SLE DAI (median, IQR) | 4.0 (2.0-6.3) | 4.0 (0.0-12.0) | 4.0 (1.0-10.0) |  | 4.0, 8.0, 9.0* |

*. All the three values were listed.
