# Supplementary material for: CD36 aggravates podocyte injury by activating NLRP3 inflammasome and inhibiting autophagy in lupus nephritis
Source: Cell Death Dis. 2022 Aug 23;13(8):729. doi: 10.1038/s41419-022-05179-9 (PMC9399182; doi:10.1038/s41419-022-05179-9)
Supplement: Supplementary file 2 — full length western blots [file 41419_2022_5179_MOESM2_ESM.docx]

**Figure2G**


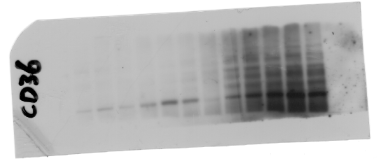


**CD36**


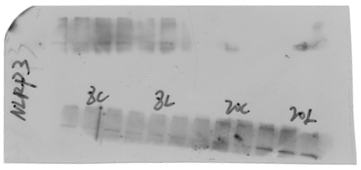


**NLRP3**

**Caspase1**


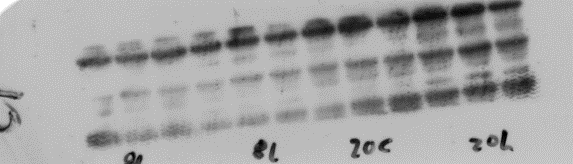


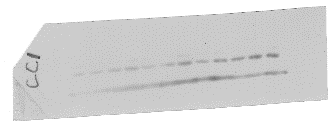


**Cleaved- Caspase1**


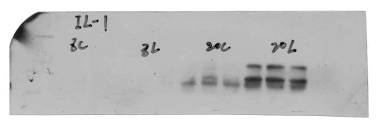


**IL-1β**


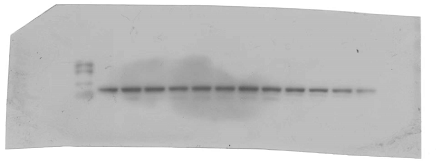


**nephrin**


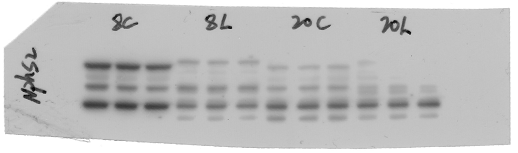


**podocin**


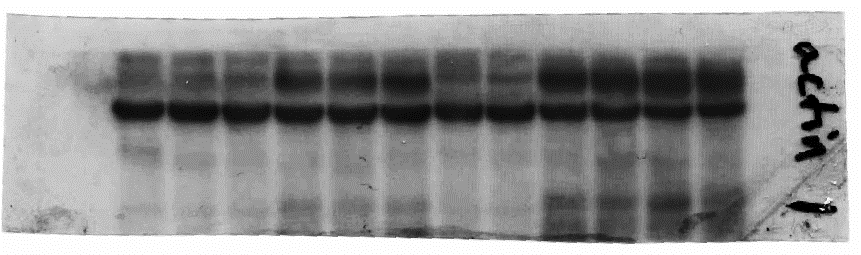


**β-Actin**

**Figure3A IgG-Control**


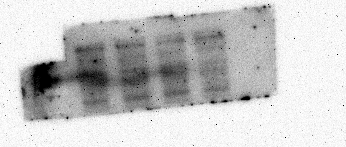


**CD36**


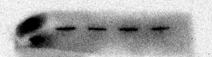


**nephrin**


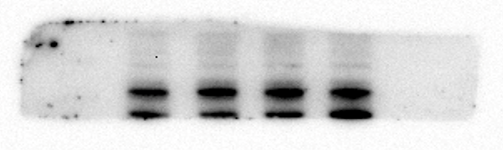


**podocin**


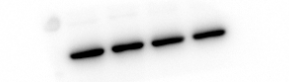


**β-Actin**

**Figure3A IgG-LN**


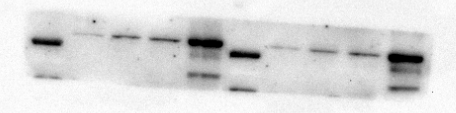


**CD36**


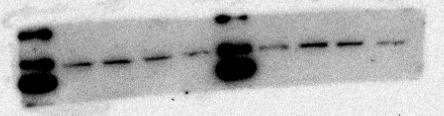

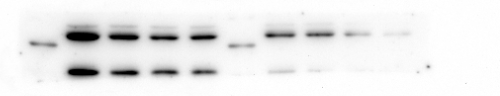

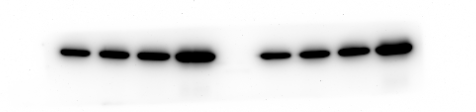


**β-Actin**

**podocin**

**nephrin**

**Figure3B IgG-Control**


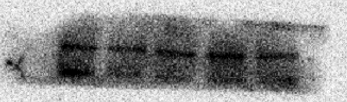


**CD36**


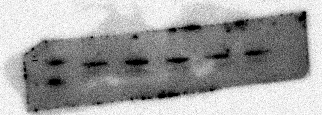


**nephrin**


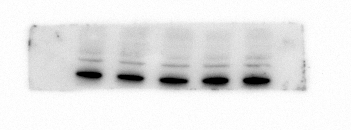


**podocin**


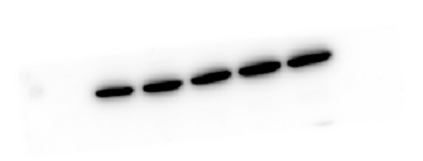


**β-Actin**

**Figure3B IgG-LN**


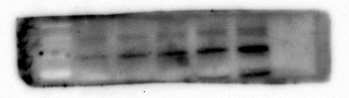


**CD36**

**nephrin**


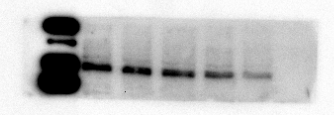

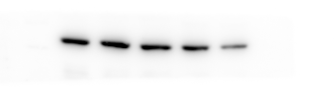

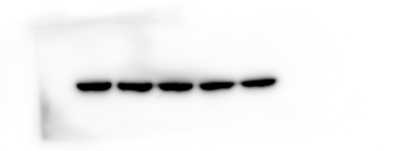


**podocin**

**β-Actin**

**Figure3C**


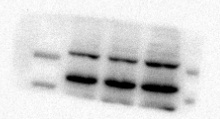


**NLRP3**

**CD36**


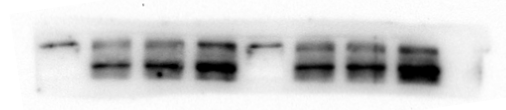


**Caspase1**


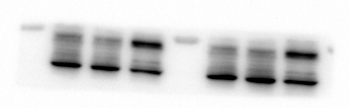


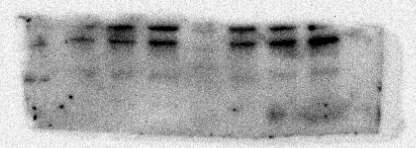


**Cleaved- Caspase1**

**IL-1β**


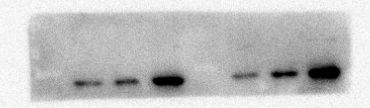


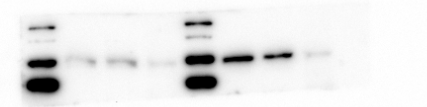


**nephrin**


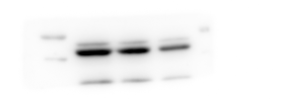


**podocin**


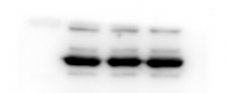


**β-Actin**

**Figure3E**


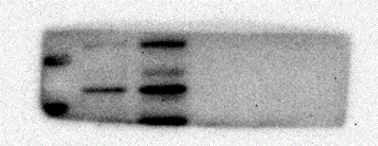


**nephrin**

**CD36**


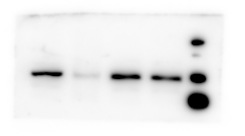


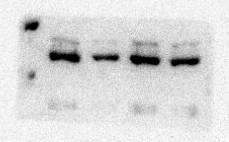


**podocin**


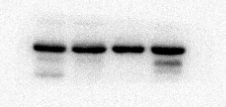


**β-Actin**

**Figure3G**


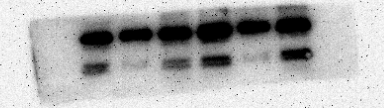


**CD36**


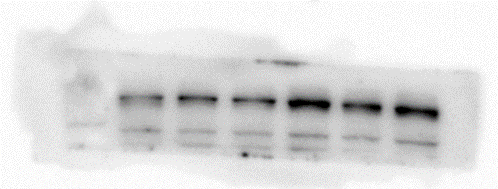


**NLRP3**


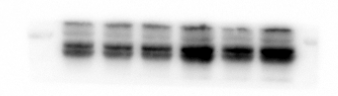


**Caspase1**


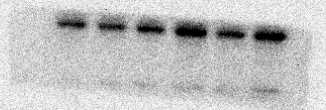


**Cleaved-Caspase1**


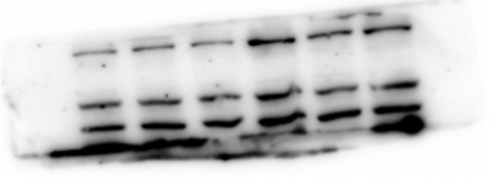


**IL-1β**


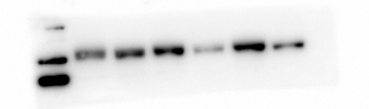


**nephrin**


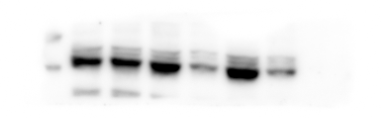


**podocin**


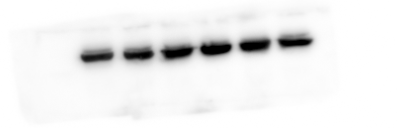


**β-Actin**

**Figure4A**


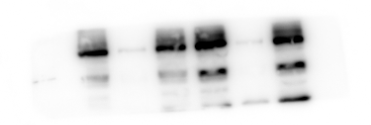


**CD36**


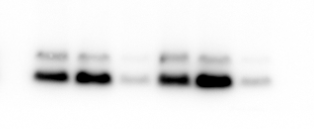


**LC3B**


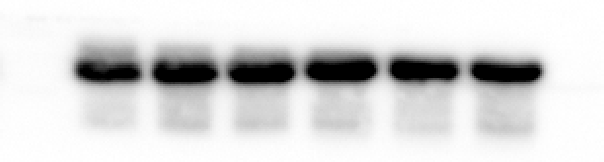


**β-Actin**

**Figure4B**


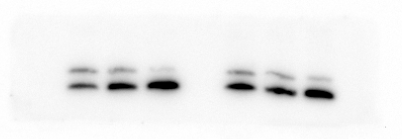


**LC3B**


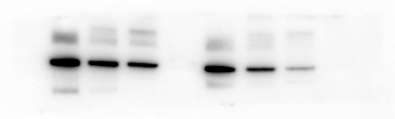


**p62**


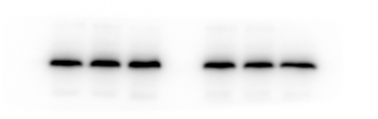


**β-Actin**

**Figure4C**


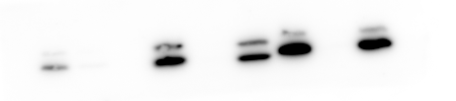


**LC3B**


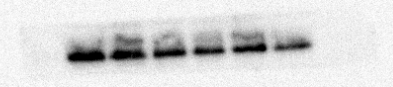


**p62**


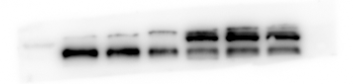


**NLRP3**


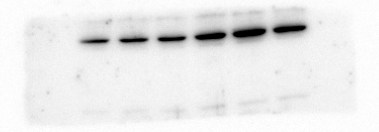


**Caspase1**


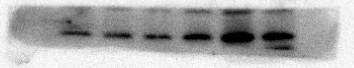


**Cleaved-Caspase1**


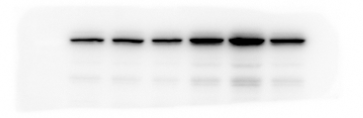


**IL-1β**


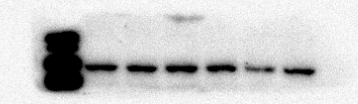


**nephrin**


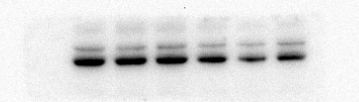

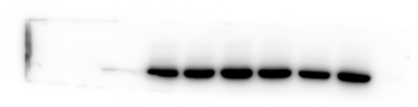


**β-Actin**

**podocin**

**Figure4D**


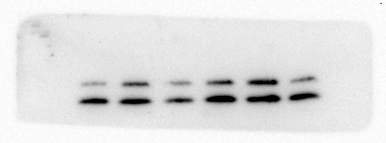

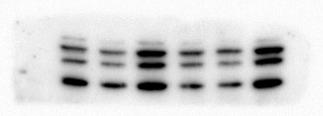


**p62**

**LC3B**


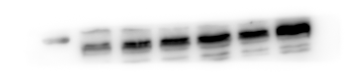

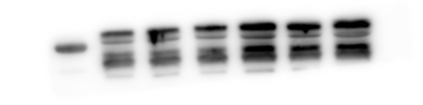


**Caspase1**

**NLRP3**


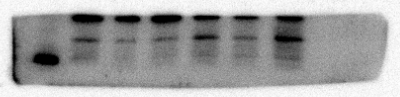


**Cleaved-Caspase1**


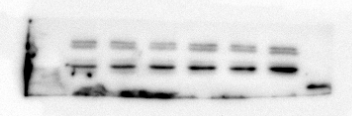


**IL-1β**


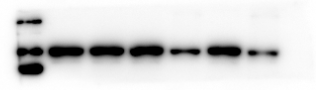


**nephrin**


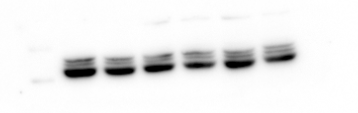


**podocin**


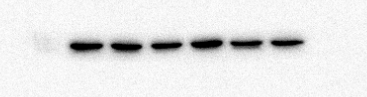


**β-Actin**
